# Acceptance of Anxiety through Art Therapy: A Case Report Exploring How Anthroposophic Art Therapy Addresses Emotion Regulation and Executive Functioning

**DOI:** 10.1155/2019/4875381

**Published:** 2019-12-27

**Authors:** A. C. Abbing, E. W. Baars, O. Van Haastrecht, A. S. Ponstein

**Affiliations:** ^1^Faculty of Health, University of Applied Sciences Leiden, Leiden, Netherlands; ^2^Clinical Neurodevelopmental Sciences, Faculty of Social Sciences, Leiden University, Leiden, Netherlands

## Abstract

Anxiety is a major problem for many individuals, causing impairment in daily life. Art therapy is often deployed and although positive results are communicated in clinical practice, its effectiveness and working mechanisms have hardly been studied. Therefore, it is important to systematically describe the intervention process and to detect the working mechanisms to be able to evaluate them. Narrative case studies help to understand the intervention in more depth. A typical case file was selected for case reporting according to scientific (CARE & CARE-AAT) guidelines, with the aim to explore the therapeutic elements that contributed to the reduction of anxiety. The report describes the intervention process of a 54-year-old female, suffering from anxiety since childhood and diagnosed with panic disorder, agoraphobia, claustrophobia and hypochondria. After 14 sessions of anthroposophic art therapy, reduction of anxiety was shown, as well as improvements of emotion regulation and executive functioning. The client indicated that she became more tolerant and accepting towards her anxiety. She noted a softened attitude towards herself and her complaints, even one year after art therapy. The course of treatment suggests that aspects of emotion regulation and executive functioning were addressed through implicit learning processes in different art therapy assignments.

## 1. Introduction

Anxiety disorders are one of the most common mental problems in the world [1] and are characterized by convulsive patterns of taking and keeping control over life situations. The Diagnostic and Statistical Manual of Mental Disorders (DSM-5) [2] distinguishes different types of anxiety disorders. The most common disorders are phobias, social anxiety disorder, generalized anxiety disorder and panic disorder [3]. People with a panic disorder experience recurrent and unexpected panic attacks, which can occur without or with a trigger, like a feared object or situation. Panic attacks, the worry about panic attacks and the effort to avoid attacks, by avoiding places, situations or behaviors, cause significant problems in various areas of daily life. This may e.g. include the development of agoraphobia: fear for situations outside the home, where leaving might be difficult or impossible [3].

Spontaneous recovery of anxiety disorders is rare [4]. However, patients may benefit from treatment. Frequently provided and well-studied interventions are pharmacotherapy (PT) and Cognitive Behavioral Therapy (CBT). CBT aims to change maladaptive beliefs about the probability and magnitude of the anticipated harms by using behavioral (exposure) and various cognitive (e.g. altering dysfunctional thoughts) techniques [5, 6]. Despite the proven effectiveness of PT and CBT [5, 7–9], an estimated 30–60% of patients do not benefit from these interventions and continue to suffer from anxiety after treatment [10–15]. Additionally, some people do not want to choose these types of interventions. Therefore, other interventions are deployed. One of these interventions is art therapy. Art therapy uses visual art exercises to elicit experiences and new insights and beliefs, with the aim to stimulate personal development and improve mental health [16]. A recent systematic review concluded that strong evidence of effectiveness for art therapy in the treatment of anxiety in adults is lacking, due to the fact that very few effectiveness studies have been performed and that the methodological quality of these studies is low [17].

Art therapy has a variety of subtypes, that are based on various perspectives from psychoanalysis, cognitive-analytic therapies, compassion-focused therapy, attachment-based psychotherapy and client-centered approaches, like mindfulness and mentalization-based treatments [16]. One of the subtypes is anthroposophic art therapy (AAT). Anthroposophic art therapists work from a holistic vision on humans [18]. The central point in this approach is that the therapist does not focus on the primary symptoms of a person, but considers the individual as a whole of physical, psycho-social and biographical aspects. The therapy is aimed at gaining insight into the processes underlying the primary symptoms and aims to initiate a holistic healing process in which the client is enabled to actively work on his or her wellbeing, including the reduction of primary symptoms [18].

A common view on people with anxiety is that they are overwhelmed by their emotions because they cannot neutralize the emotions with helpful thoughts and the mind is in a hyper-alert state [19]. Worry and rumination are often present in individuals with anxiety [2], which anthroposophic art therapists characterize as “a dominance of excessive and unproductive thinking”, which should be reduced in therapy. According to Borkovec [20] (as cited in Dar & Iqbal [21]), worrying and verbal activity interferes with emotional processing and prevents adaptive coping in individuals with anxiety.

An explicit goal of AAT is that the anxiety is not “consciously” or cognitively addressed as this will keep patients in their “thinking-mode”, enabling worry and rumination. AAT, applied through specific art assignments, is addressing the unconscious. AAT aims for the profound experience of e.g. color, atmosphere, shape and dynamics as in impressionistic art. These experiences are referred to as “impressions” in AAT and are believed to improve the self-regulating ability of the client [22–24].

Although AAT is used in Western society and positively evaluated clinically, hardly any research has been conducted to validate its mode of action. To date, one RCT on the effectiveness of AAT in women with anxiety disorders has been performed. The results are promising and show a significant reduction in level of experienced anxiety, compared to a waiting list condition [25]. From this study possible working mechanisms emerged: AAT may act through improvements in emotion regulation (strategies) and through improvement of executive functioning in daily behavior.

The aim was to gain more insight in the AAT therapeutic process leading to the reduction of experience of anxiety and supposed improved emotion regulation and executive functioning. A case of a 54-old female is described in detail. AAT therapeutic elements, and possible connections between these elements and improvements of emotion regulation and executive functioning were explored.

*Methods.* The CARE-AAT Guideline [26], which is the CARE Guideline [27] with additional categories for AAT, was used for reporting the present case. The case description is based on a case file filled by a therapist according to the CARE-AAT documentation method [28]. This information was supplemented with information from semi-structured interviews with both the therapist and the client. The interviews with the therapist were performed to fully understand the therapeutic choices that were made during the process. The interviews with the client were meant to gain insight in experiences with the art exercises, the therapist and possible changes that she experienced in health, wellbeing and daily life. The information that was gathered after the process is given below in italics and between brackets. Information meant to give background information and or insight in the reasoning of the therapist is presented in italics.

The client agreed to participate in the anonymized description of her treatment. She signed an informed consent to approve of the collection of information during art therapy and approved of publication of this article.

## 2. Case Presentation

### 2.1. Client Information

The case concerns a 54-year-old Dutch woman (referred to as Dewi (Dewi is a fictitious name)). Dewi is highly educated and works as an official at a municipality in the Netherlands. She lives together with her husband and their two children. She is neat and punctual (in clothing and time).

Dewi experienced a stressful period around the age of five or six when she was intentionally locked-up in a closet by her elder brothers/sisters multiple times. Symptoms of anxiety started to occur in her early twenties when she experienced a panic attack in a train. Subsequently, she developed fear of being trapped in trains. This fear expanded to fear of driving a car, fear of elevators and fear of losing orientation when walking outside alone. She successively tried to cope with her anxiety by allowing herself time outs, the use of slow trains, and asking somebody to accompany her whilst driving, but symptoms remained. Over the last 15 years, Dewi developed fear for becoming ill and not being able to care for her children.

A few years after the onset of anxiety attacks, Dewi received rational emotive therapy (Table 1, timeline). This did not relieve symptoms. Four years ago, she received psychotherapy with EMDR. This resulted in more insight in the cause of her anxiety but did not reduce the symptoms.

In 2017 she volunteered for AAT. At the onset of therapy, she suffered from self-diagnosed claustrophobia (especially in trains, cars and elevators), tension (distress) and hypochondria. She looked for relief of complaints, but had no specific expectations of AAT.

### 2.2. Clinical Findings and Diagnostic Assessment

Symptoms of psychopathology were assessed prior to AAT using the Dutch version of the rater-administered Mini International Neuropsychiatric Interview Plus (MINI-Plus) [29]. Dewi met the criteria for panic disorder, agoraphobia and claustrophobia. She also had symptoms of hypochondria, but did not fully meet the criteria for this classification.

The Dutch versions of the Lehrer Woolfolk Anxiety Symptom Questionnaire (LWASQ) [30, 31], the MANchester Short Assessment of QoL (MANSA) [32, 33], the Difficulties in ER Scale (DERS) [34] and the Behavior Rating Inventory of Executive Function for Adults (BRIEF-A) [35, 36] were used to assess her psychological functioning at baseline (Table 2). The scores showed high levels of anxiety (LWASQ) and severe difficulties with ER (DERS). Quality of life (MANSA) was scored as moderate and daily EF (BRIEF-A) was in the clinical range.

None of these findings were shared with the art therapist as to not to interfere with the usual therapeutic process (without formal assessment of psychological functioning). The therapist only knew the three complaints Dewi mentioned: anxiety, tension and hypochondria. During AAT the severity of these complaints were weekly scored using visual analogue scales (VAS) [37] (Figure 8).

### 2.3. Treatment

The first session served as an intake and the final session was used for evaluation. The 14 weekly sessions took place during a six-month period and were approximately 1 hour in length. Due to summer holidays AAT was discontinued for 6 weeks. Dewi did not receive other treatments during AAT.

#### 2.3.1. Treatment Goals

In the first session personal and medium specific information was collected. In the first half of the session the therapist verbally assessed symptoms and raised questions about medical, family, and psychosocial history, to gain comprehension of her symptoms and background. This was followed by a “free painting”, for diagnostic purposes [38, 39]. The aquarelle painting technique was used and ready-to-use suspensions of the primary colors in water were provided. No further instructions were given.

Dewi painted quietly and attentively. Although she worked in silence, the therapist noticed that the assignment made her a little uncomfortable. *[Dewi experienced the assignment as unstructured and too free, which made her feel uncomfortable and a bit insecure.] *The therapist decided to give another exercise.

*For the second exercise, the therapist wanted to employ a less demanding assignment. She chose for clay modelling of a sphere. Working with clay addresses the tactile sense. *Dewi appeared more familiar with clay as she had molded busts and abstract forms before and enjoyed the modeling. She indicated that she preferred modeling to painting.

Dewi's symptoms (claustrophobia and agoraphobia) indicate that her relation and interaction with the outside world is a source of distress to her. The painting shows precisely and carefully painted (curved) stripes (Figure 8). *This was interpreted by the therapist as reflecting a “controlled way of painting” which may indicate dominance of “thinking” or a hyper-alert cognitive state of mind, often seen in anxious people [19, 40]. The therapist concluded that the hyper-alert cognitive schemas needed therapeutic attention. Hyper-alertness is related to high levels of arousal [19] and high levels of emotional intensity [41]. The view in AAT is that this intensity can be diminished by relative easy assignments allowing for relaxation and by development of the skill to observe objectively. The latter can be trained by working from the observation.*

*Anxiety can also be characterized by negative affect [42]. People with high levels of negative affect tend to focus on the unpleasant aspects of themselves and the world, have negative expectations of the future and of other people [43, 44]. Developing a positive relationship with the outside world was therefore also chosen as a treatment goal.*

Based on the foregoing, the therapist set the following treatment goals for AAT:

1. Enhancing (inner) relaxation,
2. Releasing control mode and hyper-alert cognitive schemas,
3. Improving the objective observation of the outside world, and
4. Enhancing the (positive) interaction with the outside world.

#### 2.3.2. Treatment Plan

The therapist anticipated that Dewi would enjoy relatively simple and explicitly outlined art assignments with clay. This approach was chosen at the beginning of the process to allow for inner relaxation (treatment goal 1) and an appreciative feelings towards AAT and the therapist.

Then, the therapist planned to address treatment goals 2 and 3 by using precise, slightly challenging assignments with charcoal. *This material is not suitable for detailed work and may thereby stimulate loose of mental control. Charcoal also allows for the exploration of the world of greys, which thereby may stimulate the inner experience (impression) of grades of light and dark as opposed to the less nuanced mental judgements that anxious people generally have [45]*. The therapist did not plan for the explicit expression of emotions.

In the final phase, the therapist planned to bridge the return to a colorful daily life and to enhance positive feelings towards the outside world (treatment goal 4). For this purpose, soft pastels were to be used, *as they allow for the experience of both color and tactile sense as pastel chalk is wiped out with the fingertips.*

In the process the therapist wanted to exploit an encouraging and supporting therapeutic attitude. Open for questions and actively interested in Dewi's thoughts and feelings. The therapist would exploit a silent presence during the sessions to enable Dewi to experiment, practice and experience. *Too much talking can stand in the way of experiencing.* Explanations with respect to the choice of material and assignments were not given as the therapist wanted to avoid Dewi to become (hyper-)alert and take conscious control over the therapeutic process. Thus, the therapist replied to Dewi's questions about this matter in terms of: “this will become clear in a later phase” or “the experience is more important than the explanation”.

### 2.4. Therapeutic Intervention

In the *second session*, again a sphere was molded (image not shown). *This served as a comforting assignment as Dewi had done this before.* Next, Dewi was invited to transform the sphere into another form. She was asked to hold the sphere in her left hand, whilst gently transforming it with her right hand near her heart. *In this way, you cannot see what you are doing and you are mainly dependent on touch/tactile senses. This is believed to diminish mental control in the modeling process [46]*. Emphasis was given to enjoy and experience the modeling process rather than to aim for a specific clay form.

**Figure 2:**
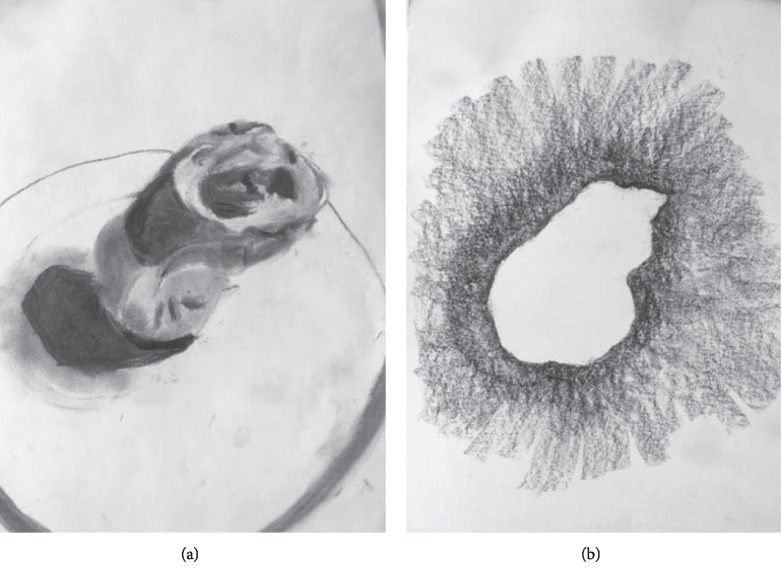
Charcoal drawings of the form modelled during session 2 and 3. Emphasis was given to the presence of light and dark, proportions and the correct representation thereof (a) and the surroundings of the form.

At the end of the session, the modeled form was drawn from observation. Charcoal was used and emphasis was given to the correct representation of the form, in terms of proportions and shades of light and dark. *The aim of this exercise was to (partly) release hyper-alertness by requesting to work in in generalities: not looking for details but concentrating on the presence of light and dark areas in forms, as shapes of their own. *The therapist provided technical support to obtain the correct proportions and a sense of 3D by exploiting the continuum between black and white (Figure 2).

In the *third session,* the modeled form of session 2 was used again as the object to draw. This time it was to be drawn “in negative”: only the surroundings of the form were to be drawn (Figure 2). *Thus, a different way of looking at the form/surroundings compared to the previous time was requested. This was done both to give Dewi comfort (by using known material and her own art work) and to challenge her (by using a new/different way of looking at her modeled form).* Dewi drew quietly.

Then an abstract drawing was made. Dewi was invited to draw several randomly positioned squares of various proportions with charcoal. The squares were to be blackened. Then, two connecting lines were to be drawn between adjacent squares. Finally, the connecting planes were to be filled with a continuous gradient from black to white [47].

**Figure 3:**
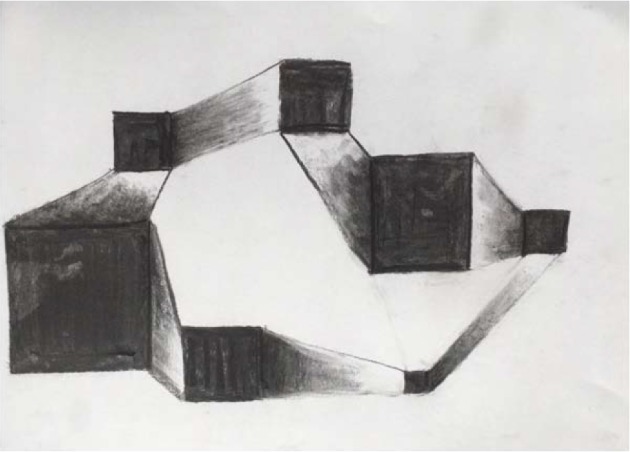
Charcoal drawing of interconnected squares. Emphasis was given to darkening of the squares and the gradual enlightenment of the connections between adjacent squares.

Dewi liked to work with charcoal and devoted herself to the proper execution of the assignment. She succeeded in gradually changing the dark into the light (Figure 3). Charcoal was on her hands and lower arms—much in contrast with her neat and punctual appearance. *The therapist interpreted this as that Dewi released some of her control and hyper-alertness, intensely connected with the charcoal drawing and became in a flow state of mind.*

At the end of the session the upcoming summer break was brought up. The therapist asked if Dewi was interested in suggestions for artistic exercises at home. Upon approval, she suggested to continue drawing from observation (and emphasize the correct representation of size, proportions and tones of light and dark) using charcoal, as in therapy. Also, the copying of black and white portrait photographs was suggested and finally, the use of soft pastels in copying impressionistic art (e.g. Monet) was mentioned.

In the *fourth session* copying of a portrait photograph (from a newspaper) was performed to illustrate the earlier suggestion for the summer break. *Dewi was reassured and challenged as well during the art work: the use of known material and working from observation were to comfort her. Copying the photograph in upside-down position was meant to challenge her and to build further on the process of correct observation.* The therapist again de-emphasized convulsive detailed drawing and stimulated the observation and representation of the larger dark and light areas in the portrait and shades of grey.

**Figure 4:**
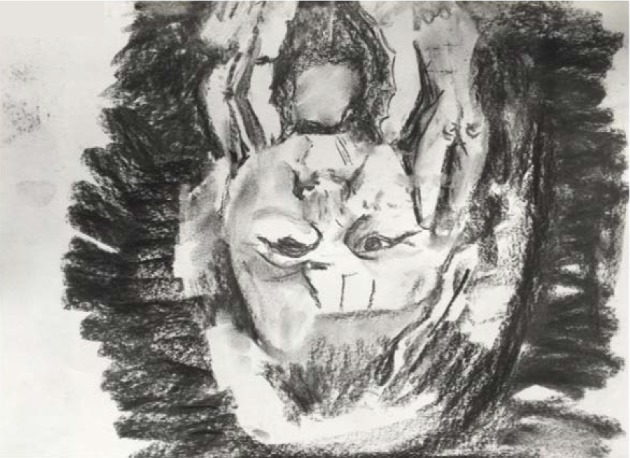
Charcoal copy of a photograph. Emphasis was given to the correct observation by turning the photograph upside down.

At the end of the drawing process the drawing (Figure 4) was turned and compared to the original. Dewi was thrilled to learn that the copy drawing looked like the original photograph.

A summer break of six weeks followed. Dewi bought some art materials and practiced at home with drawing from observation (emphasizing on the correct representation of size, proportions and tones of light and dark) using charcoal or—in case of copying impressionistic art—soft pastels.

The therapy was resumed in the *fifth session* with a basic exercise in the field of black and white encounters [48]. Dewi was invited to darken the bottom of a drawing paper with charcoal and to “dissolve” the dark into the light whilst working upwards. The dark bottom was to be convex in nature. *This was done to give Dewi the (unconscious) feeling of being supported (like a mother holding her child), to further build on a feeling of safety and create circumstances to experience comfort and warmth that may lead to (inner) relaxation*.

Dewi devoted herself to the art work, intensely and silently. It was difficult for her to create a black, convex bottom. What happened emotionally? *The therapist could not get insight in Dewi's inner experiences. She records that she had the impulse to raise questions (which she had not had before), but that Dewi's answers were unclear and not fluently given as if she rejected to the conversation.* Time was too short to finish the drawing.

**Figure 5:**
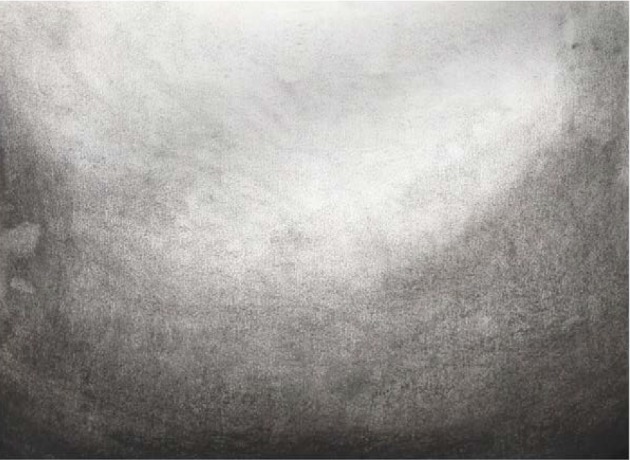
Charcoal drawing. Emphasis was given to the fluent change in darkness from the convex bottom to the top.

The *sixth session *was used to finalize the drawing. As in the previous session Dewi experienced difficulties in drawing the dark bottom and diminishing the dark fluently into the light (Figure 5). *Again, it was difficult for the therapist to get an idea of the inner process Dewi went through as Dewi did not voluntarily express her feelings and poorly answered questions raised by the therapist.*

At the end of the sixth session the therapist noticed that Dewi succeeded in the assignment and appeared calm and relaxed. *This was interpreted as that Dewi was ready for a next step in therapy. The therapist decided to address the period with anxious events (at the age of 5-6), although without the intention to verbally discuss what had happened. She rather wished to give Dewi consciousness of positive emotions that were present in that period as well and to allow her to re-engage with the anxiety at the same time. The re-engagement was to take place in a safe, supporting, accepting (therapeutic) environment and was aiming for unconscious processing. *The therapist asked Dewi to bring a photograph of herself at the age of 5-6 to the next session.

Dewi indeed brought a photograph to the *seventh session*. Without explicitly focusing on the feelings and experiences, Dewi was invited to copy (and enlarge) the photograph using charcoal. She was invited to start by darkening the whole drawing paper. Then, she was instructed to erase the charcoal in correspondence with the bright spots and planes in the photograph with an eraser. *Both the use of charcoal and the copying process were familiar to Dewi and were meant to be comforting. Dewi's attention was withdrawn from possible negative feelings by using a novel demanding drawing technique.Through art work one can distance from memories and emotions, in this case because one focusses on the observation of lighter and darker areas in the picture and tries to copy that as accurate as possible. In this process, it is possible to process earlier experiences and to gain a different connection with her childhood, e.g. more empathic, mild and understanding.*

The exercise was technically challenging. In the beginning Dewi was unable to concentrate and did not know how to start. The therapist guided and assisted her. She led her through the correct observation of planes and shades in the photograph and towards the correct representation of young Dewi. The support helped her to overcome her initial reservation and to engage in the exercise. Slowly, she understood the novel drawing process and started “to draw with an eraser”. She worked quietly. *The therapist experienced Dewi's unspoken wish not to be disturbed whilst working. *At the end of the session Dewi spontaneously told that she experienced focus and flow during charcoal drawing. In accordance, the therapist noted that negative emotions diminished during the art work.

**Figure 6:**
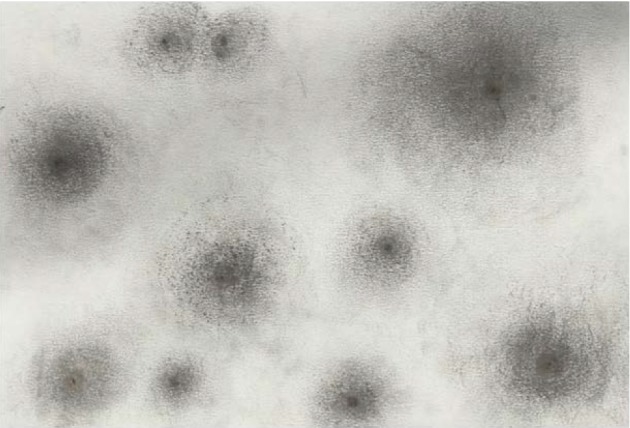
Charcoal assignment. Black dots were placed randomly on a sheet of paper and were faded out to the periphery (van den Berg, 2007).

The copy of the photograph was not finalized by the end of the session (Image not available) and had to be finished in the *eighth session*. However, Dewi forgot to bring the original. Hence, an alternative charcoal assignment was performed to further practice shading (Figure 6).

In the *ninth session* Dewi continued the copying process of the photograph. She was technically more capable of doing so. She succeeded in bringing nuances in the dark and became happy and proud about the result and her own drawing skills (image not shown upon request of the client). Inner feelings were not explicitly expressed, and no conscious attention was paid to the shocking events in early childhood either. However, the therapist noted that Dewi was emotionally at ease. At the end of the session Dewi comfortably spoke about the importance of being courageous in life.

The therapist then wanted to address Dewi's anxiety further and invited her in the *tenth session* to envision a cave. She verbally described a cave and the opening of the cave through which light from the outside world entered, and how this results in different tones of darkness in the cave. She invited Dewi to draw this mental picture using charcoal for the inside of the cave and soft pastels for the outside world (treatment goal 4: positive connection to the outside world). She also presented Dewi with photographs from the inside of caves to exemplify the changing light intensity.

**Figure 7:**
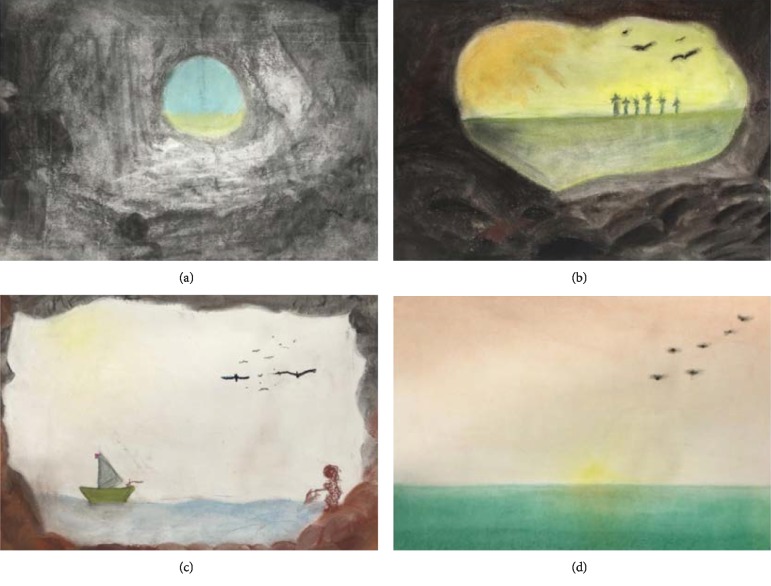
Drawings of a cave/outside world using charcoal and soft pastels. The cave (a) was drawn according to Dewi's own imagination in all drawings. In (b), trees are visible at the horizon and birds in the sky. In (c) Dewi drew a fisherman's wife on the right-hand side and in (d) a sunset in the sea is visible.

During the art work, attention was fully focused on the transition of darkness (inside the cave) to light (nearer the opening of the cave). *Dewi made the dark-light transition before and in this way, the assignment was comforting to her. The theme of the drawing (experiencing the cave as a safe place and looking to (and later engaging in) the outside world) was meant to (unconsciously) invite Dewi to positively engage with the world and in life *[49]. The outside world appeared calm and still (Figure 7(a)).

In the *eleventh session* Dewi was invited to further explore the cave and the opening to the outside world by moving towards the cave entrance. Again, charcoal and soft pastels were used and photographs of caves were presented. *Dewi could decide how fast she wanted to move from the depth of the cave to the opening. The therapist followed her pace,* encouraging Dewi to look around in her cave and depict exactly what she saw. The transition of dark into light imposed some problems, likewise, the correct use of perspective. The therapist gave technical support to overcome these issues.

The outside world looked warm and inviting as trees appeared at the horizon and birds flew in the sky (Figure 7(b)). Dewi's cave opening was larger than before, but she herself was still inside the cave (barely visible). In the *twelfth session* Dewi was invited to further explore the cave and the outside world by moving closer to the cave entrance (or even beyond). Again, charcoal and soft pastels were used and problems with the dark-light transition and perspective were met. During the session Dewi's attention changed from the inside to the outside world (Figure 7(c)). A relatively calm, bright and inviting outside world emerged. Dewi enjoyed the art work and appeared relaxed.

In the *thirteenth session* Dewi was invited to draw the outside world with soft pastels. Attention was directed to the change of color (loss of intensity) towards the horizon. In addition, she focused on the reflection of the sun light in the water. She enjoyed herself and admired the drawing and the outside world she created, its space and its calmness (Figure 7(d)).

The *fourteenth and final session* was used to evaluate the therapeutic process. The therapist prepared an exposition of all the art work as a concrete starting point for reflection.

Dewi looked back at a process that she experienced as helpful. Working with charcoal had been new to her, but a great experience. She liked to draw and became calm doing so. Art therapy had become a time and place to experience inner peace for her. She had worked with clay, charcoal and soft pastels at home but noted that that was more difficult than during sessions of art therapy.

### 2.5. Follow-Up and Outcomes

#### 2.5.1. Weekly Scores of Main Complaints

**Figure 8:**
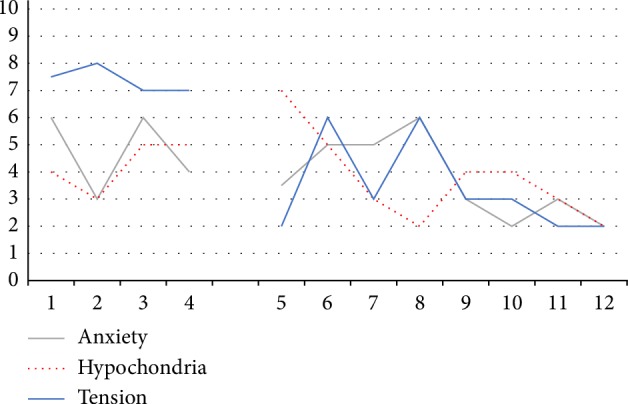
Weekly scores of Dewi's major complaints in time. Dewi gave (VAS) scores at the beginning of every session with respect to the mean experienced levels of anxiety, tension and hypochondria the week before scoring. 0: not present, 10: highest level thinkable.

Levels of anxiety, hypochondria and tension were measured at the beginning of each therapeutic session using VAS scales. Average scores for the week were given (Figure 8). Anxiety was moderately severe at the beginning (6) and fluctuated between sessions 3 and 6. In the final weeks of AAT anxiety diminished to mild (2-3).

Hypochondria increased after the first two sessions and dropped after the fifth to a level slightly lower than at the start. Tension gradually decreased from severe (7-8) to mild (2-3).

#### 2.5.2. Outcomes of Anxiety, Quality of Life, ER and EF

**Figure 9:**
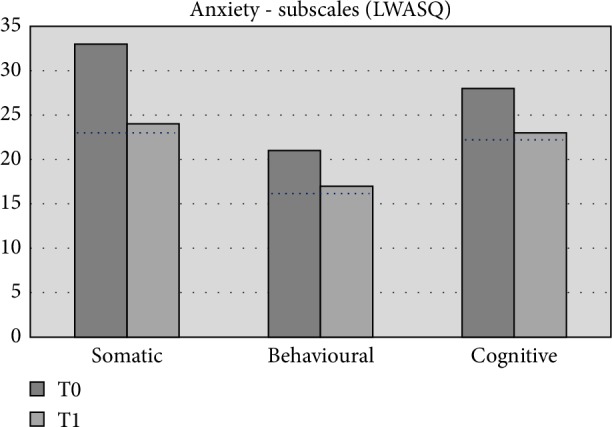
Outcomes of anxiety. Scores of Dewi are shown at T0, prior to therapy; and T1, after art therapy. The dotted black line represents norm scores in healthy population (*n* = 103) [31].

After AAT (T1) the same measures as those prior to AAT (T0) were used to quantify symptom severity (Table 2). A clear decrease in anxiety symptom severity (LWASQ) is shown. All subscales (somatic, behavioral and cognitive) showed clinically relevant improvements and approach norm scores after therapy (T1) (Table 2, Figure 9). Subjective quality of life (MANSA) improved by 2 points. As a difference of at least 4 points is considered to be a reliable improvement [50], it can be concluded that the experienced quality of life was apparently not influenced during therapy.

**Figure 10:**
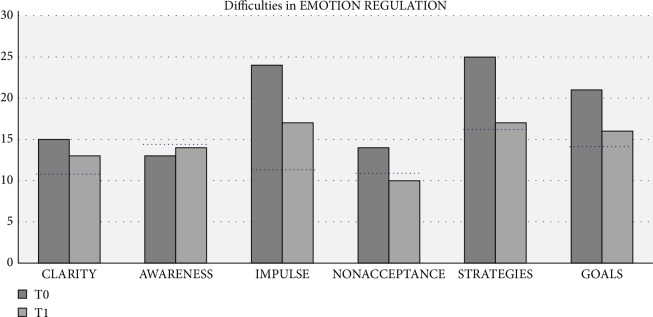
Subscales of DERS. Scores of Dewi are shown at T0, prior to therapy; and T1, after art therapy. The dotted black line represents norm scores in female population (*n* = 260) [34].

Dewi experienced less difficulties in ER after AAT. The outcomes indicate improvements in the subscales *clarity of emotions*, *impulse control*, *acceptance of emotions*, *access ER strategies* and *goal oriented action*. *Access to ER strategies* improved the most (Table 2, Figure 10).

The total score of daily behavioral EF improved, but remained in the clinical range. All domains of EF improved, except for *task evaluation* which did not change (Table 2). *Inhibit* and *self-monitor* improved from subclinical to normal scores. *Shift *and *emotion control* improved but were already in the normal range. All other domains (*initiate, working memory, plan/organize *and* organization of materials*) improved, but these scores remained in the clinical range.

#### 2.5.3. Therapist Perspective on Outcome

The therapist noted, next to the above-mentioned themes, that the interaction between Dewi and herself became more and more relaxed during therapy. She is quite confident about the positive effect of the art work on Dewi's health status both from her own observation and Dewi's verbal reflections on the therapy, both in the sessions and the evaluation.

#### 2.5.4. Client Perspective on Outcome

About four months after therapy Dewi was interviewed by one of the researchers. The intensity of her anxiety was reduced. She was still claustrophobic, but was better able to handle it. Hypochondria had improved by 70–80%, in her own opinion.

With respect to art work she noted that she had overcome her initial resistance of not being able to draw. She had had some experience with clay work and sculpting, but not with drawing. She enjoyed *“playing with light and dark”* with charcoal. She found it hard to work with her own childhood photo. It brought back feelings of loneliness that she had experienced at that age. But “*by drawing it, you distance yourself from it, from the feelings and the meaning. You look at it and draw that*.”

She had experienced AAT as very pleasant. Working on the art assignments stopped her thoughts. To be present in the moment made her experience peace and concentration, “*it was almost as meditating, and it helped me to relax. In this way it contributed to soften my feelings towards my anxiety”.*

Moreover, the different way of looking at/observing reality, as experienced during AAT, had given her the insight that it is a choice to focus on the shadow instead of the light. In daily life she spent less time anticipating for threatening situations than before AAT. She became less evasive for confrontations with fear or panic.

She experienced AAT as demanding but in a completely different way than verbal therapies:

* “Verbal therapy is hard work. You must dig into yourself so much. You do not always feel like opening up, sometimes you just do not feel like it, or it is quite tough. With art therapy it is also hard work, but in a different way. You can lose yourself for a moment in what you are doing. Art therapy, in my opinion, addressed relaxation. I was not really concerned with my emotions, but much more focused on “making it flow”.”*

AAT did not demand to explore emotions, did not request correct wording, but gave a sphere of serenity and comforting silence in the presence of a caring, well prepared, to the point and clear therapist, according to Dewi.

A year later (March 2019), Dewi was interviewed again and she indicated that, looking back, her total attitude towards her anxiety had softened. “*I really saw a development in the drawings, working towards something. Working towards myself (child photo), recalling moments. As if it opened. Just like the end of a tunnel. It felt like softening. And cheerful, light-weighted. That there was light at the end of the tunnel. Softening to myself, less hardness to myself. Maybe the fear is not gone and it never goes away completely, but that's okay. Everyone has something, I can take the time. I experienced relaxation through realizing that.”*

The claustrophobic complaints are still present, but to a limited extent. Her judgement about her anxiety changed into a more lenient attitude towards herself and her anxiety, which makes it less stressful and easier to cope with. The hypochondria symptoms are sometimes present, but less overwhelming than before. Sometimes the symptoms occur and then she goes to her GP for reassurance. Also here, she is more able to put experiences in perspective and to accept emotions as being temporarily. She can easily talk about her process and sharing with her husband and other people has proven to help. She asks for support when needed.

#### 2.5.5. Connection of Therapeutic Elements to Improvements of ER and EF

The careful, thorough and empathetic observation of Dewi and the trained individual-oriented decision-making by the art therapist is key to the design of the therapeutic process. The most prominent improvements were for ER, of which *acceptance of emotions, access to ER strategies *and *goal-oriented action* approached the norm scores (Table 2, Figure 10). EF scores were lower after therapy but remained overall in the clinical range, except for *inhibition* and *self-monitoring* which showed clinically relevant improvements. The therapist did not explicitly aim for either of the outcomes.

Based on the data, we explore now how the therapeutic elements (techniques, material and or specific assignments) may have contributed to the observed effects. With respect to ER the question is: how did art work improve* goal-oriented action, access to ER strategies *and *acceptance of emotions*?

*Goal-oriented action *is probably trained as clear goals were set by the therapist at the beginning of all sessions. The art work focused, amongst others, on achieving the goal and had to be performed in a pre-determined way. It is assumed that this unconscious addressing of goal-oriented action during art work improved goal-oriented action in through analogue processes [51] the experience of working towards a goal was applied to other situations in life. Analogy is thought to occur between the non-verbal processes in the visual medium and non-verbal psychological regulatory processes [52].

The same applies for *access to ER strategies*. This is, in essence, the demand for a multitude of ways to respond to a certain situation. In this case, it is implicitly addressed by the multitude of ways to work with charcoal and the different types of assignments (working to the observation, the personal photograph; abstract work and visionary work). The experience of using charcoal in different ways may unconsciously enhance flexibility and creativity in other life situations. *Acceptance of emotions* has probably received (un) conscious attention in sessions 7 and 9 and in the cave series (Figures 7(a)–7(d)). Although it was not explicitly mentioned, it seems likely that Dewi experienced several emotions in the indicated sessions. By focusing on the art work she was able to support herself and accept her emotions and experience that she was able to do so.

The content of the therapeutic process can also be linked to improvements in EF. The subscales *inhibit* and *self-monitor* showed improvements from (sub)clinical range to normal. Overall, specific skills were practiced during the artistic exercises. These exercises were not only intended to provide experiences and insights within a safe environment, but also to induce skill development: e.g. practicing observation, concentration and restraint. *Inhibition* is presumably trained in the various assignments in which gradual changes from black to white were explicitly made (Figures 3, 5, and 6) and the various assignments that aimed for the correct presentation of reality (Figures 2 and 4 and the personal photograph). These exercises require focus and inhibition of impulses, because strict rules must be followed to accomplish the art exercise. Dewi indeed experienced focus and concentration during the assignments. *Self-monitor *is defined as the ability to keep track of the effect of one's own behavior on other people. A connection between working on this skill and the content of the therapeutic process is not obvious in the description of the sessions, and therefore cannot be explained based on the data.

#### 2.5.6. Conclusions on Outcomes

The therapeutic process consisted of a specific series of technical steps and assignments that were thought to be both therapeutically meaningful and interesting (comforting and challenging in an acceptable balance) to Dewi, to allow her to accomplish the tasks and gain self-confidence. The consequent use of charcoal was meant to give support and a feeling of safety (predictability). The different techniques were chosen to challenge Dewi leading to a feeling of confidence when mastered. The treatment goals were not verbally or consciously addressed, but implicitly through the art assignments.

The first treatment goal—enhancing (inner) relaxation—was achieved. Dewi indicated in the interviews that she experienced peace and relaxation during art work, and relaxation in general. The second treatment goal—releasing control mode/hyper-alert cognitive schemas—seems to be achieved as well, because Dewi indicated that she could lose herself in the art work. The third and fourth treatment goals - improving the objective observation of the outside world and enhancing the (positive) interaction with the outside world—were achieved through (subconscious) training of objective observation skills during art work and the case series respectively. Drawing to the observation improves the objective observation in art work and, by an analogous process, in everyday life. In this case a more objective observation of situations causing panic possibly leads to more confidence that these situations can be handled and consequently to a more open mind towards other potentially anxious situations and subsequent behavior. The outcomes of the DERS show improvement of *acceptance of emotions* into the normal score range.

## 3. Discussion

The aim of this case report was to describe a typical AAT intervention process and to explore connections between therapeutic elements and improvements of ER and EF, both contributing to reduction of anxiety symptom severity.

The specific case of Dewi suggests that AAT resulted in anxiety symptom reduction and improved ER and EF. The description of the process, combined with the client perspective indicates that Dewi was treated in a safe and supporting environment allowing for relaxation and pleasure during art work whilst using and improving ER skills and EF. The description of the process illustrates that this learning process happened subconsciously (implicit) and not through conscious processes as in CBT.

Anxiety is known to be associated with poor ER [41, 53–55]. Improving ER is connected to reduction of anxiety symptoms [56]. ER can be explicit or implicit [57]. The explicit process, demanding conscious effort and awareness [57] can be consciously influenced. AAT appears to have a different approach. The emphasis in AAT not on consciously addressing the cause(s) of the anxiety but on initiating a guided, implicit learning curve by experience. The focus is on improving implicit ER, which is thought to occur automatically in response to stimuli [57]. The stimuli in AAT are the specific art assignments, aiming for the profound experience of colors, shapes and atmospheres and dynamics.

Our hypothesis is that this implicit route towards improving ER may lead to strengthening of implicit ER skills, but may evolve into explicit ER as well, through a cognitive process that is set in motion within the client. Further studies are needed to explore these hypotheses.

It is important to note that the content of this AAT treatment process is specific for this one client. Exercises are not to be generalized to other cases with comparable complaints by (untrained) therapists as differences between clients with respect to personality, personal experiences, coping strategies, comorbidity, social support, personal preferences for materials and assignments will influence the effect of each AAT exercise.

To further substantiate the role of the therapeutic elements that were identified in this case, more case reports are to be studied. This will lead to more insight in the relations between art work and alterations in aspects of ER and EF and hence to a better understanding of the mechanisms, and thus the art therapy specific factors by which the therapy exerts its effects. Thus, the construct of AAT will become more clear and comprehensible, and subsequently the specific factors can be tested in effectiveness studies.

Future studies should also be aimed at exploring the possibility of determining a more standardized protocol for this complex intervention (standardizing the function and process of the intervention, rather than the components) [58], that can be used in efficacy studies. More case reports and focus groups with expert therapists can contribute to this aim.

### 3.1. Strengths and Limitations

This case report is the first to describe an AAT treatment process based on prospective data collection and according to scientific case report guidelines [26, 27]. It provides detailed insight in the treatment process of AAT for anxiety, for the first time supplemented by outcomes of pre- and post-measurements, and an exploration of possible connections between therapeutic elements and improvements of ER and EF.

The quantification of complaints allowed for the comparison of the severity of complaints before and after AAT and thus for a more precise judgement of therapeutic effects than earlier AAT case reports (e.g. [59, 60]). The quantification of ER and EF opens the possibility to compare improvements with therapeutic elements (material, technique, assignment etc.) and to start studying the (or a) mode of action of AAT (2.3.6). This has not been possible before, due to the lack of data.

Unfortunately, it was not possible to create a complete description of the process. Although the therapist was requested to carefully document all stages in the process [25, 28], some information was lacking from the therapist file. For instance, arguments that were used to select the specific assignments were not provided, the professional attitude per session was not documented and notes on Dewi's daily life experiences (between sessions) were not present in the case file. It is therefore recommended to interview the therapist after each therapeutic session and or video-tape all sessions. However, this will not be possible in all cases and may also influence the normal course of therapy.

### 3.2. Takeaway Message

This case report provides insight in the route along which AAT may have led to anxiety reduction through specific art assignments. By implicitly addressing aspects of ER and EF, ER and EF were improved and anxiety symptom severity was reduced. The process by which AAT improves ER and EF may differ from the process by which verbal therapies, such as CBT, exert its actions.

Future studies should address the question whether AAT may complement or may be an alternative to CBT for specific patient subgroups, or may be suitable for patients that do not (sufficiently) respond to CBT.

## Figures and Tables

**Figure 1:**
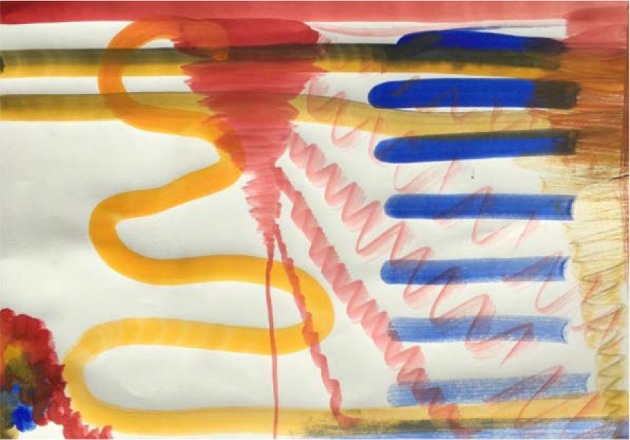
Free aquarelle painting. This painting served as the starting point for therapy.

**Table 1:** Timeline of symptoms and previous treatments.

Age	Symptoms and treatments
5/6	Anxious experiences in childhood (being frequently locked-up in a closet).
21 and beyond	Onset of panic attacks on the train, gradually increasing (only taking slow trains), expanding to fear of elevators (avoiding elevators), fear of driving (avoiding driving alone) and walking alone outside (fear of losing orientation and being lost).
23	Rational emotive therapy; no decrease of anxiety symptoms.
38 and beyond	Increase of symptoms after the birth of her children. Also, developing fear for becoming ill and not being able to care for her children.
50	Psychotherapy with EMDR: some improvements (more comprehension of the cause of the anxiety); no decrease of anxiety symptoms.
53	Applying for AAT with the following symptoms: panic attacks (fear of being locked-up and fear of losing orientation), claustrophobia and hypochondria.

**Table 2:** Outcomes of self-report measures at T0 (prior to art therapy) and T1 (after AAT).

	T0	T1	Interpretation
			Norm scores [mean (SD)] in adult population (*n* = 103)

Anxiety (LWASQ total)	**82**	**64**	**62.0 (15.9)**
Somatic (physical aspects of anxiety)	33	24	23.5 (7.1)
Behavioral (avoidance)	21	17	16.1 (6.0)
Cognitive (worry and rumination)	28	23	22.4 (6.7)

*Quality of life (MANSA)*	**63**	**65**	**64/65**

			Norm scores [mean (SD)] in female population (*n* = 260)

Difficulties in emotion regulation (DERS total)	**112**	**87**	**77.99 (20.72)**
Lack of *clarity* of emotions: the extent to which individuals know (and are clear about) the emotions they are experiencing	15	13	10.61 (3.80)
Lack of *awareness* of emotions: inattention to and lack of acknowledgement and awareness of emotional responses	13	14	14.34 (4.60)
Difficulty in controlling *impulses*: difficulties remaining in control of one's behavior when experiencing negative emotions	24	17	10.82 (4.41)
*Non-acceptance* of emotions: tendency to have negative secondary emotional responses to one's negative emotions, or non-accepting reactions to one's distress	14	10	11.65 (4.72)
Limited access to ER *strategies*: belief that there is little that can be done to regulate emotions effectively, once an individual is upset	25	17	16.16 (6.19)
Difficulties engaging in *goal*-directed action: difficulties concentrating and accomplishing tasks when experiencing negative emotion	21	16	14.41 (4.95)

Executive Functioning (BRIEF-A total)	**79**	**71**	*T-scores:*
			<60: normal range
			60–65: subclinical range
			>65: clinical range

*Inhibit*: ability to control impulses (inhibitory control) and to stop engaging in a behavior	63	55	Idem
*Shift*: cognitive flexibility, ability to move freely from one activity or situation to another; to tolerate change; to switch or alternate attention	54	46	Idem
*Emotional control*: ability to regulate emotional responses appropriately	59	54	Idem
*Self-monitor: *ability to keep track of the effect of one's own behavior on other people	65	56	Idem
*Initiate*: ability to begin an activity and to independently generate ideas or problem-solving strategies	80	70	Idem
*Working memory*: ability to hold information when completing a task, when encoding information, or when generating goals/plans in a sequential manner	85	79	Idem
*Plan/organize*: ability to anticipate future events; to set goals; to develop steps; to grasp main ideas; to organize and understand the main points in written or verbal presentations	86	80	Idem
*Organization of materials*: ability to put order in work, play and storage spaces (e.g. desks, lockers, backpacks, and bedrooms)	86	77	Idem
*Task evaluation*: ability to check work and to assess one's own performance	79	79	Idem
